# High-Grade Serous Ovarian Carcinoma Presenting With Massive Pleural Effusion in the Absence of Ascites: A Case Report and Review of the Literature

**DOI:** 10.7759/cureus.76303

**Published:** 2024-12-24

**Authors:** Sathish Krishnan, Jennifer Heisick, Melissa Johnson

**Affiliations:** 1 Pulmonary and Critical Care Medicine, Community Health Network, Indianapolis, USA

**Keywords:** indwelling pleural catheter, malignant pleural effusion, ovarian carcinoma, pleural effusion, sob - shortness of breath

## Abstract

Pleural effusion as an initial presentation of malignancy poses significant diagnostic challenges, particularly when linked to gynecologic cancers. We discuss the case of a 53-year-old female who presented with progressive dyspnea and a massive right-sided pleural effusion. Cytological analysis of the pleural fluid revealed malignant cells and immunohistochemical staining confirmed high-grade serous carcinoma (HGSC) of ovarian origin. Remarkably, there was no evidence of peritoneal carcinomatosis, ascites, or ovarian mass. PET-CT identified additional metastatic foci in the cul-de-sac. The patient was treated with systemic chemotherapy using carboplatin and paclitaxel, complemented by palliative management for recurrent effusion. This report highlights the critical importance of a multidisciplinary approach integrating clinical, pathological, and imaging findings to address atypical presentations of ovarian cancer.

## Introduction

Pleural effusion is a common clinical problem with a broad differential diagnosis, encompassing both benign and malignant etiologies. Among malignant causes, the most frequently implicated cancers include lung, breast, and hematologic malignancies such as lymphoma. Pleural fluid accumulation occurs due to pleural hyperpermeability and obstruction of lymphatic channels caused by tumor cell infiltration [1,2]. However, pleural involvement as the initial presentation of gynecologic malignancies, particularly ovarian cancer, is uncommon and often presents significant diagnostic challenges.

Ovarian cancer remains the leading cause of death among gynecologic malignancies, with high-grade serous carcinoma (HGSC) being the most prevalent histological subtype. HGSC is characterized by its aggressive behavior, early dissemination, and frequent association with advanced-stage presentation [3]. The metastatic pattern in ovarian cancer typically involves peritoneal carcinomatosis and ascites, whereas isolated pleural effusion without peritoneal involvement is rare, occurring in less than 5% of cases [4]. This atypical metastatic spread may involve hematogenous or lymphatic dissemination rather than the more common transcoelomic route [5].

Immunohistochemical and molecular profiling have emerged as critical tools in differentiating primary ovarian carcinoma from other malignancies that metastasize to the pleura. Markers such as paired box gene 8 (PAX8) and cytokeratin 7 (CK7) are frequently utilized to confirm an ovarian origin, while negative staining for markers like thyroid transcription factor 1 (TTF-1) helps exclude pulmonary primaries [6]. Additionally, elevated tumor markers such as cancer antigen-125 (CA-125) aid in diagnosis and guide therapeutic decisions.

In this report, we present the case of a 53-year-old female with metastatic HGSC of ovarian origin, initially manifesting as a massive pleural effusion. This report underscores the importance of a multidisciplinary approach and highlights the role of advanced diagnostic modalities in managing atypical presentations of ovarian cancer.

## Case presentation

A 53-year-old female presented to the emergency department with a three-week history of progressively worsening shortness of breath. Her past medical history included moderate persistent asthma, type 2 diabetes mellitus, and coronary artery disease. She also reported a worsening cough that was mildly productive of clear sputum. She denied abdominal pain, abdominal distension, dysuria, diarrhea, fever, chills, or night sweats. She was a former smoker with a 15-pack-year history. Her family history was negative for malignancy. Notably, she had undergone coronary artery bypass grafting (CABG) three years before this presentation.

**Figure 1:**
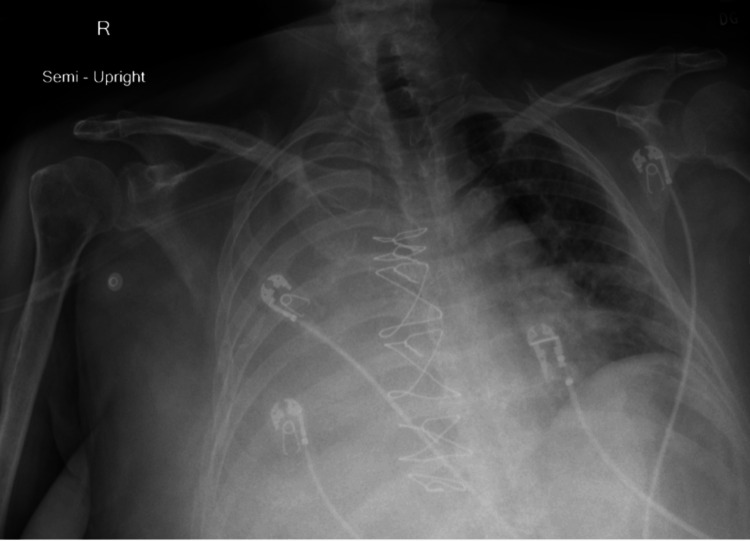
Chest radiograph of the patient The image shows complete opacification of right hemithorax, suggestive of large right pleural effusion

**Figure 2:**
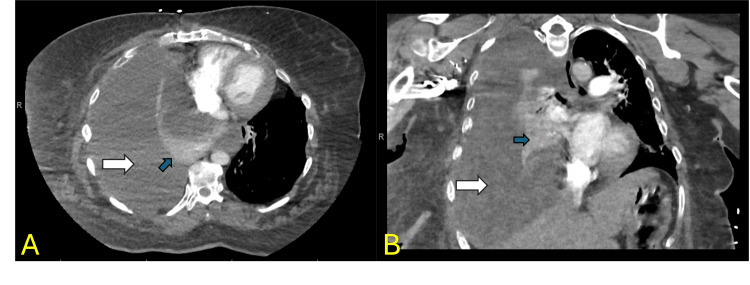
CT scan of the chest Axial section (A) and coronal section (B) show large right pleural effusion (white arrows) with complete atelectasis of the right lung (blue arrows) CT: computed tomography

On presentation, her vital signs included a temperature of 96.84 °F, a pulse rate of 84 beats per minute, blood pressure of 169/80 mmHg, respiratory rate of 34 breaths per minute, and oxygen saturation of 86% on room air. She required 2 liters of oxygen via nasal cannula to maintain oxygen saturation above 90%. A chest X-ray revealed complete opacification of the right hemithorax with a mild mediastinal shift to the left (Figure 1). Subsequent chest CT confirmed a large right pleural effusion with complete atelectasis of the right lung. No lymphadenopathy, mass, or nodule was observed (Figure 2). A diagnostic thoracentesis was performed, and 2 liters of blood-tinged fluid was drained. The pleural fluid analysis indicated an exudative effusion according to Light's criteria. Biochemical characteristics of pleural fluid and serum are summarized in Table 1 and Table 2 respectively.

**Table 1:** Pleural fluid - serum biochemistries and serum tumor marker evaluation AFP: alpha-fetoprotein; β-hCG: beta subunit of human chorionic gonadotropin; CA: cancer antigen; CEA: carcinoembryonic antigen; LDH: lactate dehydrogenase; PF: pleural fluid

Pleural fluid and serum characteristics	Patient value	Reference range
PF LDH	>900 U/L	<50% of serum level
PF protein	4.5 g/dL	1–2 g/dL
PF pH	7.34	7.6–7.64
PF glucose	143 mg/dL	65–99 mg/dL
PF white blood cell count	1194/μL	<1000/μL
PF lymphocyte percentage	60%	Not established
PF red blood cell count	11,000/μL	<100/μL
Serum LDH	200 U/L	120–246 U/L
Serum protein	6.5 g/dL	6.1–8.1 g/dL
Serum glucose	162 mg/dL	65–99 mg/dL
Tumor markers		
CA-125	76 U/mL	<35 U/mL
CEA	<2 ng/mL	<2 ng/mL
CA 19-9	17 U/mL	<35 U/mL
AFP	1.2 ng/mL	<6.1 ng/mL
β-hCG​​​	<3 mIU/mL	<5 mIU/mL

**Table 2:** Immunohistochemical findings TTF-1: thyroid transcription factor 1; PAX8: paired box gene 8; GATA3: G-A-T-A nuclear marker 3

Marker	Result
Cytokeratin 7	Strong positive
TTF-1	Negative
PAX8	Positive
Calretinin	Negative
GATA3	Negative
p53	Strong positive

Two days later, the patient experienced worsening dyspnea, and a repeat chest X-ray revealed reaccumulation of the pleural effusion with complete opacification of the right hemithorax. A chest tube was inserted for slow, complete drainage. Final pleural fluid cytology confirmed the presence of malignant cells, with immunohistochemical results consistent with HGSC. Serum tumor marker findings and the immunohistochemical results are summarized in Table 1 and Table 2 respectively.

**Figure 3:**
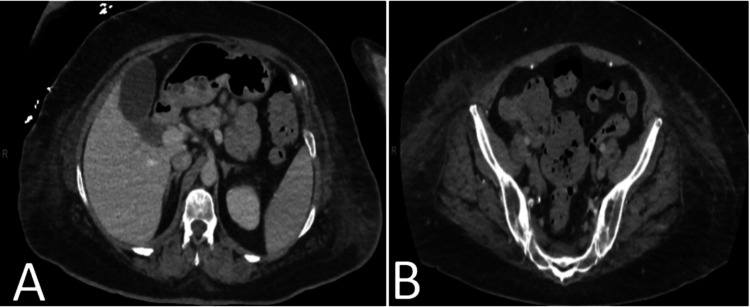
CT scan of the abdomen (A) and pelvis (B) showing no ascites CT: computed tomography

**Figure 4:**
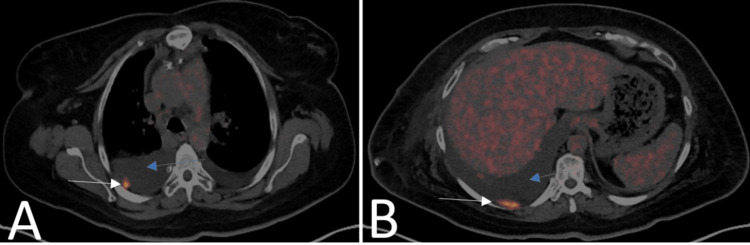
PET-CT scan of the chest The image shows multiple foci of FDG uptake in the pleura (white arrows) and pleural effusion (blue arrows) FDG: fludeoxyglucose; PET-CT: positron emission tomography-computed tomography

**Figure 5:**
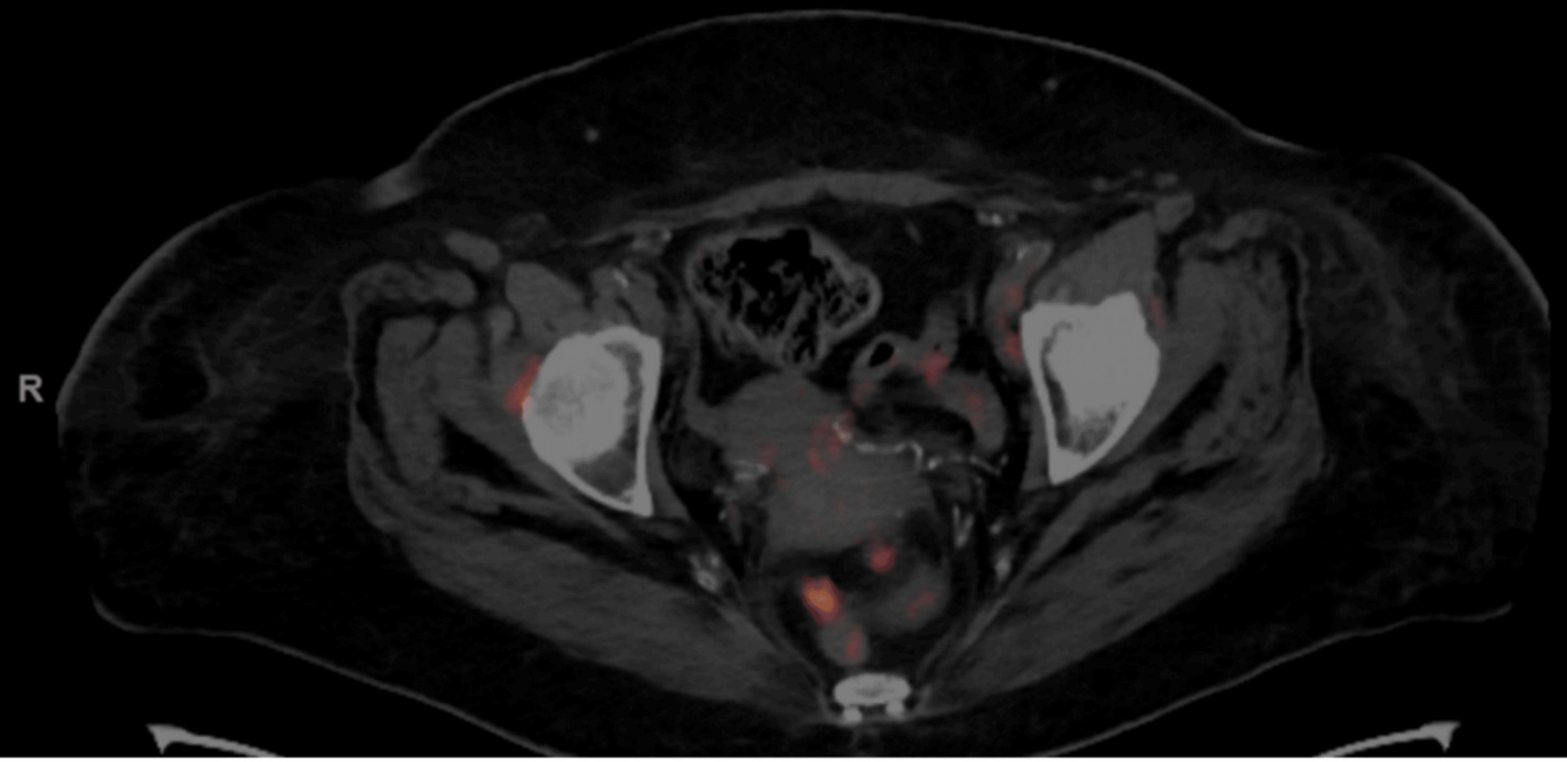
PET-CT scan of the pelvis The image shows two foci of abnormal soft tissue and increased FDG uptake in the cul-de-sac region between the upper rectum and upper vaginal pouch, suspicious for metastatic foci PET-CT: positron emission tomography-computed tomography

Further imaging with a CT of the abdomen and pelvis with intravenous contrast revealed no abnormalities (Figure 3). Transvaginal ultrasound showed a normal uterus and right ovary, while the left was not well visualized. A trace amount of fluid was noted in the cul-de-sac. The patient also underwent a colonoscopy, which yielded normal findings. A PET-CT scan demonstrated areas of fluorodeoxyglucose (FDG) uptake in the right pleural space and two small foci of abnormal soft tissue in the cul-de-sac region (Figures 4-5).

The patient's case was reviewed at a multidisciplinary tumor board, where metastatic high-grade serous carcinoma of likely ovarian origin was confirmed. Treatment with carboplatin and paclitaxel chemotherapy was initiated. An indwelling pleural catheter was placed to manage recurrent effusions, facilitating outpatient care.

## Discussion

Pleural effusion as the initial manifestation of malignancy presents a diagnostic challenge, particularly when associated with gynecologic cancers. This case highlights an atypical presentation of HGSC, where pleural effusion appeared before significant pelvic findings. Such rare cases warrant thorough diagnostic evaluation with a high index of suspicion. Malignancies commonly associated with pleural effusion include lung, breast, and lymphoma, which account for the majority of cases [1,2].

Gynecologic malignancies, while less frequently implicated, have been known to metastasize to the pleural space, with ovarian carcinoma being the most prominent [3]. Among ovarian cancers, HGSC is the most prevalent histological subtype, characterized by aggressive behavior and a high propensity for distant metastases [4]. Ovarian cancer presenting primarily as pleural effusion, without peritoneal carcinomatosis or ascites, is an unusual clinical scenario, observed in less than 5% of cases [5]. The pathophysiology behind this phenomenon likely involves hematogenous or lymphatic spread, rather than transcoelomic dissemination, which is the more common route [6].

The diagnosis of HGSC in this patient was established by cytological examination of pleural fluid, which revealed malignant cells. Immunohistochemical staining confirmed the ovarian origin, showing strong positivity for CK7 and PAX8, with negativity for TTF-1 and GATA3. These findings are consistent with the immunoprofile of HGSC, aiding in its differentiation from primary pulmonary or breast carcinomas [7,8]. Elevated CA-125 levels further endorsed the ovarian origin of the malignancy, as this marker is frequently elevated in HGSC and serves as both a diagnostic and prognostic tool [9]. Imaging studies were notable for their subtlety. While the CT of the abdomen and pelvis did not reveal significant abnormalities, the PET-CT demonstrated FDG uptake in the pleural space and small foci in the cul-de-sac, consistent with metastatic disease. This highlights the value of advanced imaging modalities in identifying occult metastatic sites when conventional imaging is inconclusive [10,11].

The management of HGSC with pleural involvement typically involves systemic chemotherapy, with carboplatin and paclitaxel as the standard first-line agents. These drugs have shown efficacy in controlling disease and improving survival outcomes [12]. In this case, the placement of an indwelling pleural catheter provided symptomatic relief and facilitated outpatient management of the recurrent effusion. This approach is consistent with current guidelines for managing malignant pleural effusions, emphasizing both symptom palliation and quality of life [13,14].

From a prognostic standpoint, pleural involvement in HGSC is associated with advanced-stage disease and a generally poor prognosis [15]. However, early recognition and appropriate intervention can significantly impact the patient's clinical trajectory. This report underscores the importance of considering ovarian cancer in the differential diagnosis of unexplained pleural effusion, even in the absence of significant pelvic findings.

## Conclusions

This report underscores the diagnostic complexity of pleural effusion as an atypical initial presentation of HGSC of ovarian origin, particularly in the absence of peritoneal carcinomatosis and ascites. Advanced diagnostic techniques, including immunohistochemical staining, tumor marker analysis, and comprehensive imaging such as PET-CT, are essential for confirming the ovarian origin and identifying occult metastatic sites. A multidisciplinary approach that integrates these findings is crucial for accurate diagnosis and effective management. Systemic chemotherapy, combined with palliative measures such as control of malignant pleural effusion, can significantly improve patient outcomes and quality of life, highlighting the importance of vigilance in evaluating unexplained pleural effusions.
